# Typhoid Fever and Helminth Coinfection: A Pediatric Case Report

**DOI:** 10.7759/cureus.19808

**Published:** 2021-11-22

**Authors:** Nnennaya Opara

**Affiliations:** 1 Emergency Medicine, Charleston Area Medical Center (CAMC) Health Education and Research Institute, Charleston, USA

**Keywords:** constipation, abdominal pain, fever with rash, enteric fever (typhoid fever), ascariasis lumbricoides

## Abstract

Enteric fever and helminth infestation coinfection is commonly seen among children below the age of 5, living in areas with poor sanitation in Africa. These can be explained due to the fact that both enteric fever and ascariasis, are contracted via fecal-oral routes. Although the immune system of children is presumed to be stronger and capable of eliminating several infectious agents, it is not applicable to children below the age of 5. Balanced nutrition also plays a vital role in sustaining strong immunity in children of all age groups and so, it could be one of the contributing factors to high susceptibility to co-infectious diseases among children living in poor countries. Soil-transmitted helminths (STH) are very common in developing countries. They are caused by infection with roundworm, hookworm, or whipworm. Both typhoid fever and helminth infestation in children presents with almost similar clinical symptoms. We present a case of coinfection with typhoid fever caused by Salmonella typhi bacteria and helminth in a 4-year-old child from Nigeria.

## Introduction

Parasitic infestation and typhoid fever are seen in children of all ages, especially in developing countries. These infectious diseases are spread through fecal-oral routes [1]. In children, the most common agent for typhoid fever is Salmonella typhi bacteria. It is an enteric fever with main clinical symptoms such as; abdominal pain and fever. There are other serotypes of Salmonella typhi: types A, B, and C which cause similar syndromes. Typhoid fever is a multisystem public health problem, particularly in developing countries. In general, typhoid fever is caused by Salmonella typhi and Salmonella paratyphi, both members of the family Enterobacteriaceae [2-3]. Nontyphoidal salmonella (NTS) is more typical in children and is limited to gastroenteritis [3]. The term “enteric fever” is used for both infections caused by Salmonella typhi and Salmonella paratyphi. Salmonella infections are spread by houseflies, dirty hands, feces, fomites of infected patients, etc. it is only transmitted from an infected person to another person, as humans are clearly their only host. Its pathogenesis depends mainly on the infectious species, virulence, the host’s immunity, and infectious dose [4]. Fever due to typhoid comes in a stepwise pattern (high in the morning and falls in the evening) and is accompanied by headaches and abdominal pain followed by rashes around the abdomen

Helminth infestations are often referred to as neglected tropical diseases because they disproportionately affect impoverished population [5]. For this reason, very few investigations have been channeled to these topics that could be the key to solving these kinds of diseases in the endemic regions. Similarly, typhoid fever is an infectious disease that affects people of all ages in developing countries. The severity of gastroenteritis symptoms in children is explained by coinfection with enteric pathogens [6]. Acute helminth infections are also seen in asymptomatic people, which makes it difficult to link gastroenteritis to a specific etiological agent particularly in our case.

Overall, patients with coinfections are often regarded as immunocompromised, compared to patients with single infections [7]. Coinfection also can impact the growth and development of children. It also affects treatment cost, probably as a result of clinical complications with co-infecting pathogens [8]. These are the reasons for the need for more research studies aimed at investigating the role of coinfections with enteric pathogens in children.

## Case presentation

**Figure 1:**
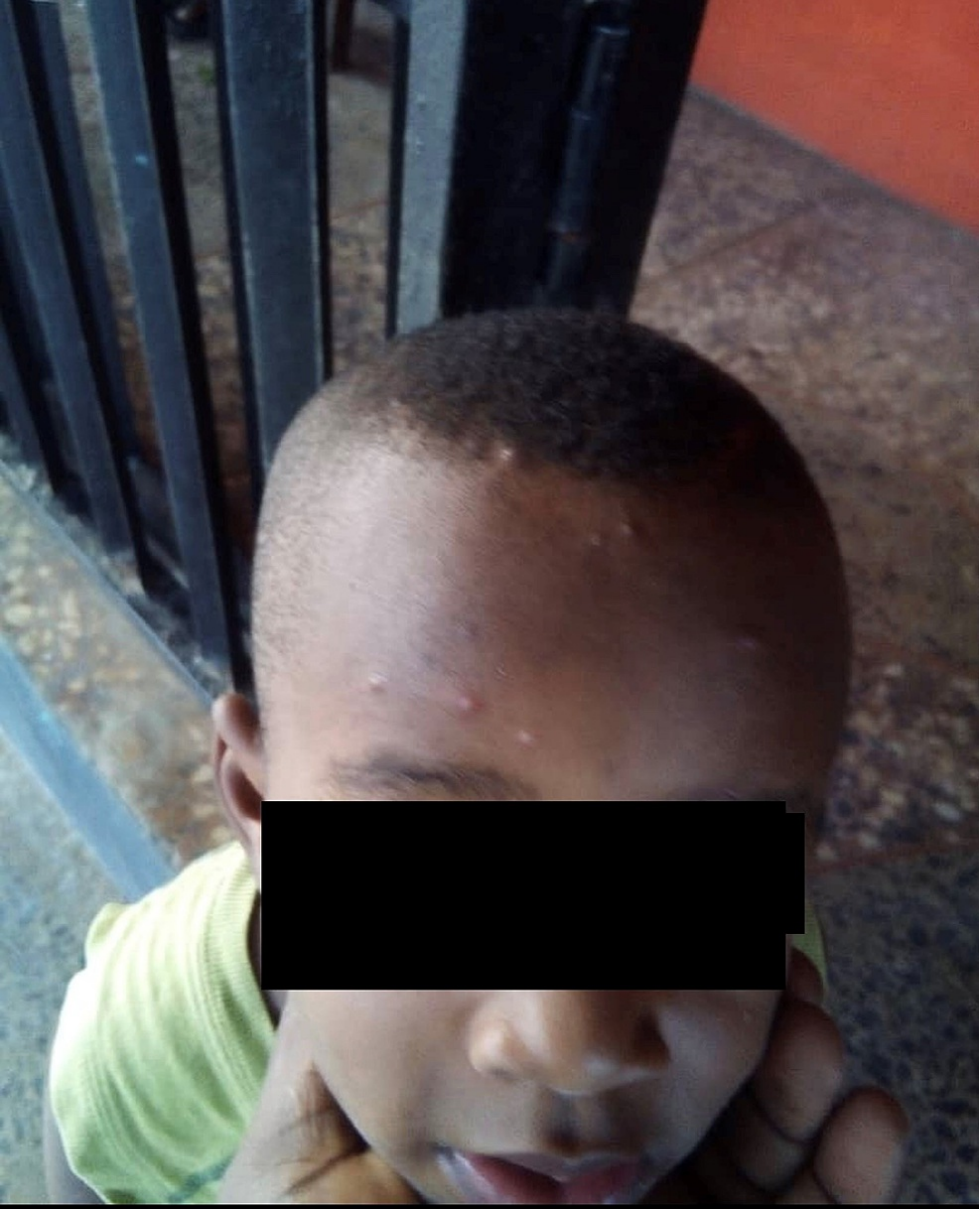
Patient with maculo-papular rashes on the head and extremities from allergic response to helminthiasis and enteric fever

This is a case of a 4-year-old male who was brought to our clinic with complaints of four-day history of constipation, dry cough, vomiting, high fever (104 °F), abdominal pain with bloating, headache, and rash. The patient’s symptoms started gradually with fatigue, loss of appetite, muscle aches, cough, bloated abdomen, and poor oral intake, prior to presenting to the hospital. The parents assumed it was stomach flu, and so managed their child’s symptoms with Tylenol and soups. However, the patient continued to have constipation, abdominal discomfort, and eventually maculo-papular rashes on the head, face, and extremities erupted (Figure 1).

**Figure 2:**
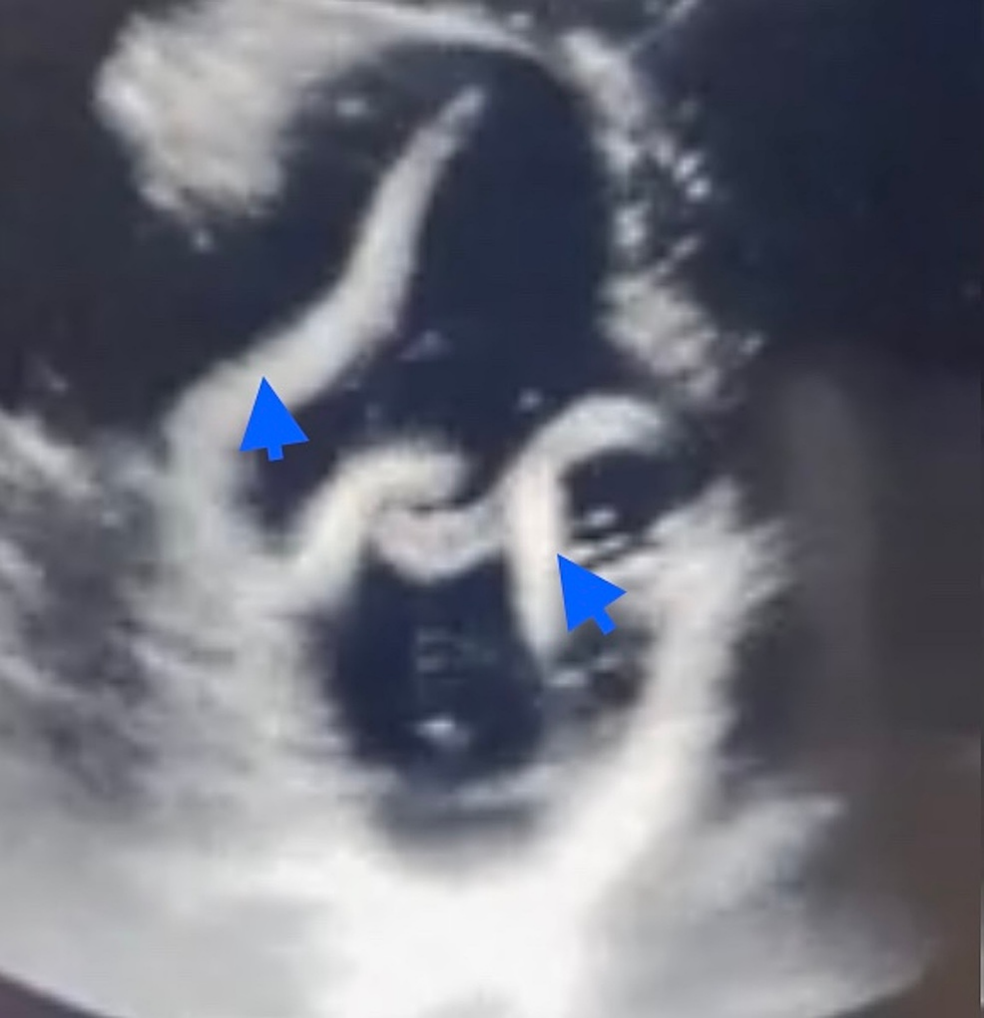
Abdominal ultrasound showing roundworms (blue arrows) in the jejunum

On day 1, upon admission to the hospital, IV fluid with 0.9% normal saline solution was started, due to signs of dehydration, bradycardia, and hypotension. Norepinephrine was also administered. Lab samples (stool, urine, and blood) were collected for analysis, and abdominal ultrasound was ordered which showed clumps of worms in the jejunum which explained the constipation our patient had (Figure 2).

Stool test for helminth (stained with bile) showed rounded 45-78 micrometer long thick-shelled eggs indicative of roundworm infestation (Ascaris lumbricoides). Blood test came back positive for typhoid DNA and increased level of eosinophils with relatively high leukocytes. The rest of the complete blood count (CBC) was normal including a chest X-ray.

Following day 2 of in-hospital admission, the patient’s condition remained unstable due to high fever (102 °F), vomiting, and fatigue. Antibiotics were initiated with ceftriaxone, antipyretics, albendazole, and more IV fluids.

Finally on day 3, the patient’s symptoms improved clinically, although body rashes persisted. CBC had normalized, and he was later discharged home with one week prescription of azithromycin and albendazole. The patient was also prescribed antihistamine cream to help stop the itching from the rash.

## Discussion

Infections with roundworms present with similar clinical features of bronchial asthma and typhoid fever, especially in pediatric patients with wheezing, cough, fever, and gastrointestinal symptoms [9]. It can easily be confused with acute bronchiolitis in children below age 5. Enteric fever in children has a more severe clinical presentation in patients with ascariasis than in those uninfected, and so the presence of roundworm coinfection compounds the well-recognized challenges of reaching a definitive diagnosis in children with suspected enteric fever [10]. Therefore, it is imperative that clinicians screen for helminths in patients with symptoms of gastrointestinal infections, particularly in patients living in endemic areas/developing countries.

Ascariasis, caused by roundworms affects over 800 million children in developing countries resulting in poor growth, impaired cognitive development, poor school attendance, and malnutrition [11]. Worms have the ability to cause damages to our internal organs from the effects of pressure exerted by growing parasites resulting in intestinal obstruction. Roundworms also cause immunopathology responses (eosinophilia, rashes) in the form of hypersensitivity reactions (pneumonitis, urticarial, petechial hemorrhages, and pruritus) as seen in our patient. When treating children in developing countries, CBC with eosinophilia, should raise suspicion for helminthiasis, as eosinophils remains essentially unchallenged as the cell at centerpiece of granulocyte-helminth correlation [11].

The overall prevalence of enteric fever-helminth coinfection in children less than 5 years of age living in Nigeria is approximately 51.14% [12]. However, such a high prevalence of coinfection among children below the age of 5 is not a shock to us clinicians because children at this age have immature immunity against enteric fever and helminths, and still possess the habits of eating the soil which may be contaminated with helminth eggs. The most effective management for both helminth infestation and enteric fever (typhoid fever) is prevention. These include safe drinking water, proper sanitation, in addition to prompt effective medical. Overall, the primary preventive measure for both people that travel to a typhoid fever endemic areas and people living in such areas, is the vaccine. The first single shot is given approximately one week before travel, and the second dose of the vaccine is given orally in four capsules which should be taken every other day [13]. Note: the vaccine efficacy does wear off over time. Thus, it requires repeat immunization when necessary.

## Conclusions

These infectious diseases could be prevented with improved sanitation and good personal hygiene - frequent hand washing, clean drinking water, consumption of cooked hot foods, and avoiding eating unwashed raw vegetables. When managing young patients from developing countries who present to the clinic with gastrointestinal syndromes, it is important to also rule out helminths infections especially among children below the age of 5. This is because helminths infestations always trigger immune responses which could lead to misdiagnosis of other potential infectious pathogens. Additionally, primary preventive measures could be the key to decreasing/eliminating the incidence of enteric fever and helminth infestations in both children and adults. So, more efforts are needed in the implementation of effective health improvement practices in the tropical regions in Africa.
